# Mushroom marvels: understanding their role in human health

**DOI:** 10.3389/fnut.2025.1654911

**Published:** 2025-08-05

**Authors:** Sana Noreen, Hazeefa Sultan, Bushra Hashmi, Patrick Maduabuchi Aja, Ayomide Victor Atoki

**Affiliations:** ^1^University Institute of Diet and Nutritional Sciences, The University of Lahore, Lahore, Punjab, Pakistan; ^2^Faculty of Biomedical Sciences, Kampala International University, Bushenyi, Uganda

**Keywords:** edible mushrooms, health benefits, nutritional value, phytochemicals, industrial usage

## Abstract

Edible mushrooms have long been valued not only for their culinary appeal but also for their significant nutritional and medicinal properties. This review examines the chemical composition, nutritional value, and bioactive potential of various edible mushroom species. Mushrooms are rich in moisture, proteins, fibers, vitamins, minerals, and a diverse array of bioactive compounds, including phenolic acids, flavonoids, sterols, polysaccharides, and terpenoids. These components confer a range of health benefits, notably antioxidant, anti-inflammatory, anti-microbial, anti-diabetic, hepatoprotective, neuroprotective, and immunomodulatory effects. Furthermore, the review emphasizes the industrial applications of mushrooms in pharmaceuticals, nutraceuticals, cosmeceuticals, agriculture, and biotechnology. Despite their benefits, certain mushroom species pose toxicological risks due to compounds like amatoxins and ibotenic acid, underscoring the importance of proper identification and consumption practices. This study aims to bridge traditional knowledge and modern scientific insights, thereby supporting the integration of mushrooms into functional foods and therapeutic regimens that enhance human health and well-being.

## 1 Introduction

A mushroom is the fleshy reproductive structure of a fungus, commonly emerging above the soil, characterized by a cap, stalk, and gills. Although some mushrooms are edible, several varieties are toxic, making accurate identification imperative. The name “mushroom” primarily denotes gilled fungi, such as the button mushroom, although it may also encompass other gilled or stemless fungi (1). *Agaricus bisporus*, commonly called mushrooms, is a member of the Agaricaceae family, exhibiting variations in shape, color, and characteristics among different species (2, 3). About 1.5 million fungi have been found, and 14,000 species of fungi can produce fruiting bodies considered as mushrooms, and only 200 species are referred to as an edible source of food (4). Most of the edible mushrooms are *Agaricus, Auricularia*, *Hericium, Pleurotus*, *Cordyceps*, *Lactarius,* and *Pisolithus* (5). Edible mushrooms are cultivated and grown from soil, trees, plant cuttings, or seeds. The duration of mushroom development varies among species; some, such as morels, require an extended period to mature, while others, like oysters, grow rapidly. The extensive cultivation of edible mushrooms is prevalent globally (6). The global consumption of mushrooms as a food source is prevalent. These are consumed for their delectable flavor and superior nutritional content (7, 8). The nutritional makeup of mushrooms consists of 85–95% moisture, 3–5% protein, 0.5% fat, and 6–10.9% minerals. Edible mushroom is an excellent source of essential amino acids, including leucine, glutamic acid, aspartic acid, valine, and glutamine. Besides this, the protein content in edible mushrooms significantly contributes to promoting good health, as these mushrooms also contain nonessential amino acids, specifically gamma-aminobutyric acid (GABA), which acts as a major neurotransmitter. Various vitamins are contained in significant quantities, including niacin, riboflavin, biotin, thiamine, pantothenic acid, and folic acid. A significant quantity of minerals, notably zinc, copper, iron, and potassium, is contained in fruiting bodies and regarded as a nutritious food source (9, 10). The extract of different parts of mushrooms exerts potential biological benefits against human disorders by anti-oxidants, anti-inflammatory, anti-microbial, anti-obesity, and many other pharmacological properties. The composition of phytochemical compounds of mushrooms helps to improve and promote human health. The phenolic constituents of edible mushrooms possess the ability to reduce the risk of multiple human disorders. The phenols, bioactive compounds, enzymes, peptides, protein, and other derivatives of phytochemicals have potential against free radicals and viral agents (6, 11). Mushrooms have been valued for centuries for their possible medicinal advantages as well as for their taste. Growing scientific curiosity in recent years has brought attention to the nutritional value and bioactive substances found in different kinds. The present research on the health-promoting characteristics of mushrooms—including their involvement in enhancing immunity, supplying vital nutrients, and providing possible anticancer, antioxidant, and anti-inflammatory effects aims to be explored and synthesized in this paper Given growing worldwide focus on natural health products and functional meals, knowing how mushrooms contribute to human nutrition and health becomes very important. This study aims to close the gap between conventional wisdom and contemporary scientific discoveries by providing an understanding of how mushrooms could be successfully included in regular meals for better general health.

## 2 Chemical composition of edible mushrooms

### 2.1 Nutritional values of edible mushrooms

**Figure 1:**
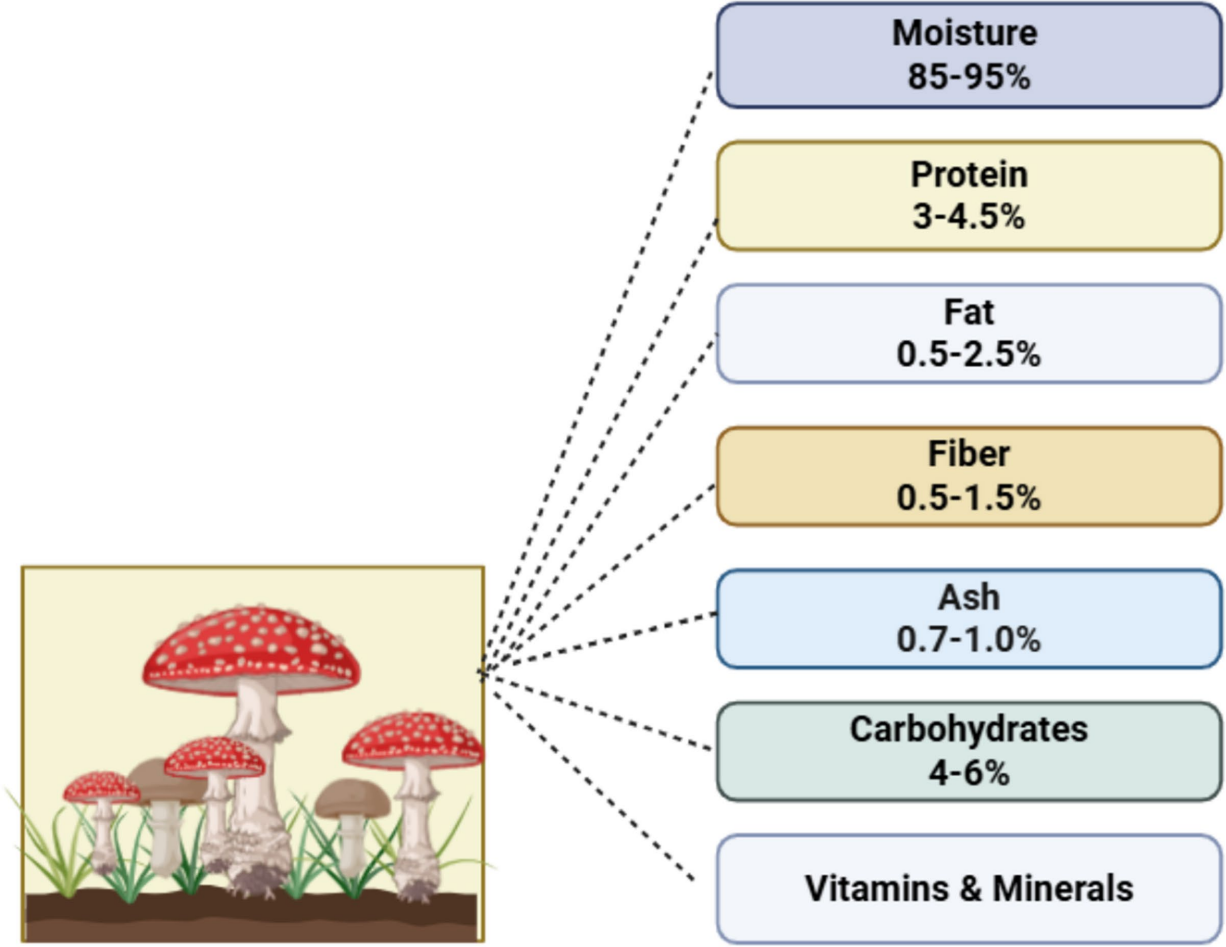
Nutritional values of edible mushrooms.

For millennia, edible mushrooms have been extensively consumed for their palatable flavor as well as their health-enhancing and therapeutic attributes (12, 13). Nutritional content in edible mushrooms includes protein, vitamins, minerals, fat-free, fiber, as shown in Figure 1. Protein in edible mushrooms plays an important role due to the presence of all essential amino acids, which are powerful components for human growth and development. It also plays a chief role in human muscle protein (14, 15). Several experimental studies proved that a wide range of carbohydrates have been found in edible mushrooms, mainly glucose and mannitol, and a minimum quantity of sucrose and fructose, also seen in the nutritional components of mushrooms. Mushrooms contain polysaccharides, including *β*-glucans like lentinan and pleuran, which are abundant in *Lentinula edodes* and *Pleurotus ostreatus*. The β-glucan content in mushrooms typically comprises 20–45% of total carbohydrate (10–25 g/100 g dry weight) in some species (16). Due high proportion of insoluble dietary fiber (22 to 30%) in edible mushrooms, it helps to prevent constipation. Besides this, edible mushrooms also comprise 4 to 9% of soluble dietary fiber, which helps to lower the cholesterol level. Various studies have demonstrated that the *Pleurotus ostreatus* mushroom contains 4.1 g of dietary fiber (17–19). In addition to nutritional value, edible mushrooms are considered as most healthy and nutritious food. The nutritional content of mushrooms varies from species to species due to different harvest stages and variations of the environment in which the species of mushrooms are grown (20). The consumption of edible mushrooms is high due to their nutritional and chemical composition importance in human dietary regimen. The oyster mushrooms have an oyster-like shape and are widely used for food due to their delicious taste, nutraceutical functions, and medicinal benefits (21). In several experimental studies, different methods of extraction were used to identify the nutritional, medicinal, and therapeutic properties of fruiting bodies like mushrooms (14, 22). They are enriched with a great source of all nutrients, but lipids comprise a low quantity in the nutritional composition of mushrooms. The amount of fat concentration in the total dry weight of edible mushroom (*Agarics bosporus*) is 1.66 to 2.2%. Also, the major fatty acids present in the mushroom contribute to the prevention of arthritis and heart-related diseases. Linoleic acid, oleic acid, and palmitic acid are found in edible mushrooms in greater quantities, which are involved in maintaining blood pressure by lowering the cholesterol level (23, 24). Linoleic acid, a polyunsaturated fatty acid found in high proportions in *Calocybe gambosa*, *Hygrophorus marzuolus*, and *Pleurotus species*, makes up 30–70% of mushroom fatty acids and 150–300 mg/g of total fat. It has significant antioxidant and anti-inflammatory properties, suppressing the synthesis of important pro-inflammatory cytokines such as interleukin-6 (IL-6), interleukin-1 (IL-1), and tumor necrosis factors (TNFs) (25). Ergosterol can also serve as a precursor for the synthesis of vitamin D in the human body. The antioxidant component tocopherols also aids in protection against the detrimental effects of free radicals and mitigates heart-related problems (26).

Several studies have also shown that edible mushrooms are a great source of all major vitamins and minerals. Vitamin B complex, including B1, B2, B3, B7, B9, B12, and vitamin D and other major minerals like iron, zinc, copper, and phosphorus, as mentioned in Table 1. Due to low levels of sodium, calories, and gluten, it has a positive impact on human health (26). Other studies showed that *Pleurotus ostreatus* has the highest concentration of vitamin B1 and B3, and ergosterol (100–500 mg/100 g dry weight) present in *Agaricus bisporus* and *Pleurotus ostreatus*. The presence of *α*-tocopherol in *Craterellus cornucopioides* and *Lactarius deliciosus* varies from 2 to 7 mg/100 g dry weight, depending on species and growth circumstances (27, 28).

**Table 1:** Edible mushroom varieties with their mineral content per 100 grams.

Varieties of mushrooms	Sodium	Potassium	Phosphorus	Magnesium	Calcium	Iron	Zinc	Selenium (μg)	References
White Button (*Agaricus bisporus*)	5.3	318.4	86.2	9.5	3.2	0.5	0.5	9.3	(3)
Cremini (Brown) (*Agaricus bisporus*)	6.4	448.4	120.3	12.6	3.1.	0.7	0.5	26.1	(28)
Portobello (*Agaricus bisporus*)	9.1	364.2	108.4	14.1	3.5	0.5	0.5	18.2	(85)
Shiitake (*Lentinula edodes*)	13.2	304.5	112.2	20.3	2.3	0.4	1.0	5.7	(11, 79)
Oyster (*Pleurotus ostreatus*)	18.3	420.2	120,3	18.4	3.4	1.3	1.3	2.6	(83)
King Oyster (*Pleurotus eryngii*)	15.4	361.1	120	12.2	3.5	1.1	1.9	3.6	(81, 83)
Knoki (*Flammulina velutipes*)	3.3	359.2	105	16.5	3.4	0.3	1.6	2.4	(47, 60)
Mitake (*Grifola frondosa*)	4.1	204.2	68	9.2	1.1	3.5	1.3	2.9	(52, 61)
Chanterelle (*Cantharellus cibarius*)	7.2	506.8	81	13.4	1.5	3.5	1.2	0.2	(20, 34)
Morel (*Morchella esculenta*)	13.9	411.3	194	19.5	4.3	12.2	2.1	2.1	(50, 87)
Lion’s Mane (*Hericium erinaceus*)	5.2	320.9	120	15.2	2.2	1.0	1,5	2.6	(81, 88)
Beech Mushroom (*Hypsizygus tessellatus*)	4.9	394.4	110	14.3	2.8	0.5	1.4	1.5	(11, 23)
Wood ear (*Auricularia auricula-judae*)	28.1	300.4	70	12.2	1.9	1.6	1.0	2.2	(79)
Paddy Straw (*Volvariella volvacea*)	12.2	260.2	120	10.2	3.5	1.9	0.9	1.9	(52)
Cauliflower (*Sparassis crispa*)	6.4	290.1	165	10.3	2.7	0.7	0.5	2.3	(1, 3, 23)

### 2.2 Functional molecules of mushrooms

**Figure 2:**
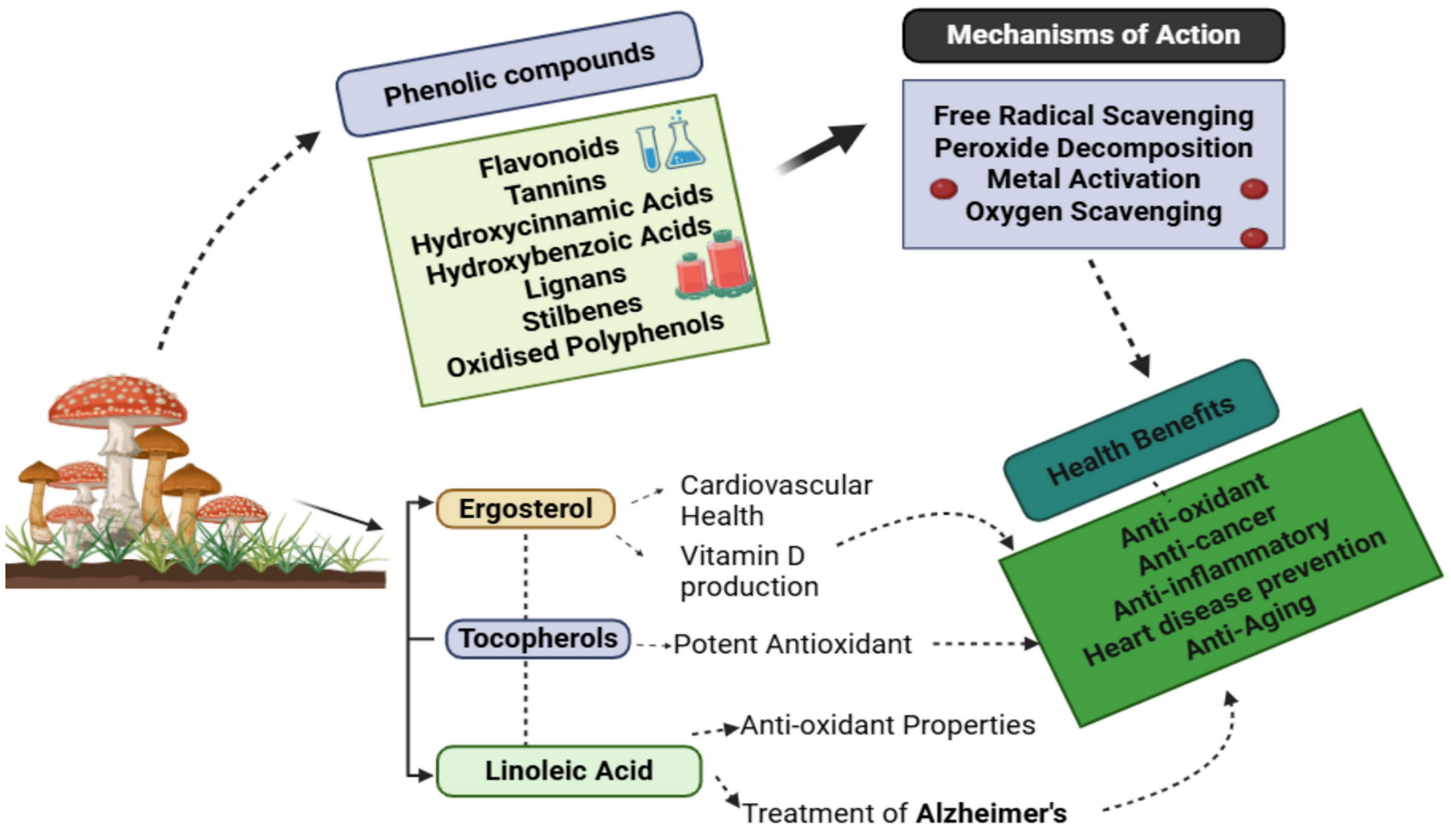
Health benefits of phenolic compounds in mushrooms.

Besides the macronutrients and micronutrients outlined in Section 2.1, mushrooms are abundant in many functional bioactive molecules, such as phenolic compounds, terpenoids, alkaloids, polysaccharides, sterols, and antioxidants. These chemicals are essential to the medicinal and therapeutic efficacy of mushrooms and enhance their health-promoting attributes. Phenolic compounds constitute a substantial class of secondary metabolites in mushrooms, playing a crucial role in their antioxidant, antibacterial, anti-inflammatory, and cytoprotective properties (Figure 2). This category includes phenolic acids, including gallic acid, protocatechuic acid, and p-hydroxybenzoic acid; flavonoids (catechin, myricetin, and quercetin); as well as tannins, lignans, and oxidized polyphenols (Table 2). Edible mushroom species such as *Pleurotus ostreatus*, *Calocybe gambosa*, *Hygrophorus marzuolus*, *Agaricus bisporus*, *Lactarius deliciosus*, and *Boletus edulis* have total phenolic levels between 1 and 6 mg/g dry weight, but flavonoid concentrations often range from 0.9–3.0 mg/g (27). Ellagitannins and lignans have been found in *Boletus edulis* and *Lactarius* spp. at 1–2 mg/g. These phenolic compounds neutralize free radicals, prevent lipid peroxidation, and control inflammation. Terpenoids, which come from isoprene units, are one of the most active types of chemicals found in mushrooms. They are well known for their ability to protect the brain, reduce inflammation, protect the liver, and fight cancer. Hericenones and erinacines are present in *Hericium erinaceus* and help make nerve growth factor (NGF). The amount of these compounds depends on how they are extracted and can range from 1 to 10 mg/100 g dry weight. The triterpenoids are another well-known category. They also have ganoderic acids, which are present in *Ganoderma lucidum* and have properties that protect the liver, fight cancer, and change the immune system (29). Alkaloids are nitrogen-containing compounds that possess neurological, antibacterial, and adaptogenic capabilities; nevertheless, they are only found in trace levels. These alkaloids can alter the cholinergic system, which protects the brain and improves cognitive function (26). Mushrooms are high in sterols and antioxidants, making them even better for health. Ergosterol, a sterol found in mushrooms, is a precursor to vitamin D₂ and has potent anti-inflammatory, antioxidant, and anticancer effects. When mushrooms are exposed to UV rays, ergosterol transforms into vitamin D₂, increasing their nutritional value. It is present in a number of species, including *Agaricus bisporus*, *Pleurotus* spp., and *Lentinula edodes*. The amount varies from 0.5 to 5.0 mg/g dry weight, depending on the species and growing technique. Mushrooms, which contain sterols, are a good source of antioxidants in the diet (29). These include glutathione and ergothioneine. *Pleurotus eryngii*, *Agaricus bisporus*, and *Flammulina velutipes* have significant levels of ergothioneine, a sulfur-containing substance that protects cells. The amount of ergothioneine in these mushrooms varies from 0.4 to 2.0 mg/g dry weight. It aids in the elimination of reactive oxygen species and promotes mitochondrial health. Mushrooms contain glutathione, a potent antioxidant that works with ergothioneine to keep cells in redox balance, protect them from oxidative damage, and promote general cell function (30, 31). These chemicals contain antioxidant, anti-inflammatory, and immune-boosting properties and provide therapeutic advantages in avoiding or controlling various disorders, as discussed in section 3 (5, 32).

**Table 2:** Pharmacological or medicinal activities in edible mushroom species.

Mushroom species	Compounds	Phenolic compounds	Health benefits	Study type	References
*Agaricus bisporus*	Amino acids (aspartic, glutamic, histidine, lysine) Fatty acid (linoleic, palmitic) Vitamins (vitamin B complex and vitamin D)	Gallic acid, Protocatechuic acid p-hydroxybenzoic acid Vanillic acid Syringic acid	Protect against oxidative stress and microbial agents. Enhances immune defense through natural vitamin D synthesis	In vitro In animals In humans (elderly; 15–30 g/day UV-exposed mushrooms for vitamin D support)	(13, 42, 43)
*Boletus edulis*	Proteins, polysaccharides, phenolic acids, flavonoids, and total phenols ergosterol Micronutrients (zinc, selenium, potassium)	Gallic acid Ferulic acid p-coumaric acid Syringic acid Catechin Vanillic acid	Antioxidant activity Anti-bacterial Anti-viral Cholesterol-lowering potential source of bioavailable selenium	In vitro In animals In humans (selenium absorption from ~100 g cooked mushrooms)	(30, 37, 72)
*Cantharellus cibarius*	Carotenoids Polysaccharides Phenolic compounds ergosterol Tocopherols Micronutrients	Gallic acid Ferulic acid Protocatechuic acid Caffeic acid p-hydroxybenzoic acid	Antioxidant activity Anti-inflammatory Anti-viral, immunomodulatory, Support for eye and skin health Natural source of provitamin A	In vitro In animals In humans, benefits are inferred from nutrient content (provitamin A)	(34, 56)
*Coprinus comatus*	Polysaccharides, Phenolic compounds Ergothioneine Flavonoids Vitamins, Essential amino acids, Unsaturated fatty acids Trace minerals	Gallic acid, Caffeic acid p-coumaric acid Ferulic acid Syringic acid Vanillin	Hepatoprotective, Anti-diabetic, anti-microbial Anti-inflammatory Supports uric acid metabolism (gout management)	In vitro In animals In humans (approx. 1–2 g/day extract)	(18, 61)
*Cordyceps militaris*	Cordycepin (3′-deoxyadenosine) Polysaccharides Ergosterol Essential amino acids, unsaturated fatty acids Phenolic compounds and flavonoids	Gallic acid Protocatechuic acid p-coumaric acid Caffeic acid	Antimicrobial Antiinflammatory Antifatigue Anti-tumor Improves oxygen uptake and endurance	*In vitro* In animals In humans (athletes; 1–3 g/day extract for 6–12 weeks)	(38, 51, 63)
*Craterellus cornucopioides*	Polysaccharides Phenolic compound Flavonoids Essential minerals B-complex vitamins	Gallic acid Ferulic acid Vanillin Syringic acid	Immunomodulatory effects Anti-inflammatory boosts cellular immunity rare dietary source of vitamin B12 analogs	In vitro In animals	(19, 89)
*Craterellus lutescens*	Polysaccharides Phenolic acids Flavonoids Carotenoids Ergosterol Tocopherols (vitamin E) Ascorbic acid (vitamin C) Essential minerals B vitamins	Caffeic acid Ferulic acid Gallic acid p-coumaric acid	Antioxidant, anti-microbial Anti-inflammatory Immunomodulatory effects Cardiovascular support high vitamin E and C synergy Used in cancer adjuvant therapy (chemo support)	In vitro In animals	(13, 41, 47)
*Ganoderma lucidum*	Triterpenoids Polysaccharides Sterols Vitamins and minerals Phenolic compounds and flavonoids Proteins	Gallic acid Syringic acid p-coumaric acid Ferulic acid Vanillic acid	Anti-inflammatory Immunomodulatory effects Cardiovascular support Neuroprotective Antidiabetic promotes gut microbiota balance	In vitro In animals In Human (cancer/diabetes patients; 1.5–5 g/day extract)	(2, 13, 54)
*Grifola frondosa*	Sterols Phenolic compounds Flavonoids Essential amino acids, unsaturated fatty acids, and dietary fiber Vitamins and minerals	Gallic acid Protocatechuic acid P-hydroxybenzoic acid Vanillin	Anti-hypertensive activities Hepatoprotective Anti-inflammatory Immunomodulatory effects Cardiovascular support Neuroprotective Anti-diabetic	In vitro In animals In Human (diabetics; ~2.5 g/day extract for 8 weeks)	(30, 33)
*Hericium erinaceus*	Hericenones Erinacines Sterols Phenolic compounds Flavonoids Essential amino acids	Gallic acid Syringic acid Ferulic acid Caffeic acid	Anti-depression/Anti-anxiety Neuroprotective and cognitive enhancement Gastrointestinal support Neuroregenerative stimulates nerve growth factor (NGF), gut-brain axis support	In vitro In animals In Human (mild cognitive impairment; 3 g/day for 16 weeks)	(24, 52, 53)
*Lentinula edodes*	Triterpenoids Polysaccharides Sterols Vitamins and minerals Phenolic compounds and flavonoids Proteins	Gallic acid p-coumaric acid Syringic acid Ferulic acid	Anti-microbial Anti-inflammatory Immunomodulatory effects Cardiovascular support Gastrointestinal health	In vitro In animals In Human (hyperlipidemic patients; 5–10 g/day dried mushrooms)	(15, 50, 69, 79)
*Agaricus blazei*	Polysaccharides Triterpenoids Phenolic compounds Vitamins and Minerals	Protocatechuic acid Vanillin P-hydroxybenzoic acid Gallic acid	Anti-microbial Anti-cancer Immunomodulation anti-metastatic enhances NK cell activity	In vitro In animals In Human (cancer patients; ~3 g/day extract)	(9, 20)
*Amanita caesarea*	Polysaccharides Phenolic compounds Carotenoids Ergosterol Vitamins and Minerals	Gallic acid Ferulic acid p-coumaric acid	Immunomodulatory effects Cardiovascular support Anti-depression/Anti-anxiety Neuroprotective and cognitive enhancement	In vitro In animals	(12, 69)
*Marasmius oreades*	Triterpenoids Polysaccharides Sterols Vitamins and minerals Phenolic compounds and flavonoids Proteins	Gallic acid, Syringic acid Ferulic acid	Anti-cancer Immunomodulation natural antifungal activity against dermatophytes	In vitro In animals	(39, 72)
*Russula griseocarnosa*	Polysaccharides Sterols Vitamins and minerals Phenolic compounds	Gallic acid Caffeic acid Vanillic acid	Anti-microbial Immunomodulatory effects Cardiovascular support	In vitro only	(11, 44)
*Sanghuangporus sanghuang*	Triterpenoids Nucleosides Polysaccharides Sterols Vitamins and minerals Phenolic compounds	Gallic acid Syringic acid p-Hydroxybenzoic acid Caffeic acid	Anti-Arthritis, Anti-bronchitis, Anti-hypertension, Anti-gastric ulcer Anti-inflammatory Immunomodulatory effects	In vitro In animals	(57, 90)
*Ganoderma applanatum*	Essential amino acids Polysaccharides Sterols Vitamins and minerals	Gallic Acid Vanillin Protocatechuic acid	Antibacterial activity Antitumor Antiproliferative activity	In vitro In animals	(11, 79)
*Morchella elata*	Polysaccharides Phenolic compounds Fatty acids Amino acids Vitamins and minerals	Gallic acid p-hydroxybenzoic acid Vanillic acid	Antioxidant Anti-microbial Immunomodulation Hepatoprotective Nephroprotective effects	In vitro In animals	(21, 52)
*Pleurotus citrinopileatus*	Triterpenoids Polysaccharides Sterols Vitamins and minerals Phenolic compounds and flavonoids Proteins	Gallic acid Syringic Acid Ferulic Acid Caffeic Acid	Antioxidant Anti-cancer Immunomodulation Gastrointestinal health	In vitro, In animals	(50, 79, 91)

## 3 Mushrooms as bioactive functional food ingredients

Mushrooms have significant value in food processing due to their high nutritional and functional properties that enhance the quality of the final product. During the development process of food products, ingredients are considered fundamental for manufacturing products in the food industry (33). Although ingredients offer the opportunity to enhance the quality and nutritional characteristics of processed foods. The food products made from natural ingredients, obtained from different food sources, are safe to eat and prevent various diet-related diseases (34, 35). According to the Food and Drug Administration, Mushrooms are enriched with substances that contribute to promoting the nutritional profile of products directly or indirectly. The nutraceutical property of edible mushrooms makes them more useful in the production of dietary supplements, nutraceuticals, and functional foods. The high amount of all-important nutrients in mushrooms fulfills the nutritional requirements of the human body, and it’s used as a dietary supplement alternative to various food sources. Agro-wastes and agro-industrial wastes have been utilized by the mushrooms and provide a good source of protein (36, 37). During food production, packaging, and storage, proper management and use of ingredients play an important role in all stages of food processing. The modification of ingredients to improve their texture, appearance, and taste attracts the attention of consumers and increases the acceptability of the product. Ingredients are also considered nutraceutical as they possess the potential to prevent various diseases and provide several human health benefits (38, 39).

Mushrooms have the potential to prevent physiological conditions and provide biological functionalities to human health. Generally, mushrooms possess a savory taste due to the existence of sodium salt of glutamic and aspartic; therefore, these mushrooms have a palatable taste and are called umami. The overall taste of mushrooms is enhanced by the presence of amino acids like monosodium glutamate (13, 40). Due to their unique flavor, mushrooms are recommended for the functional and nutraceutical food composition. Mushrooms’ umami taste has boosted their quality and has been used to produce delectable foods (41, 42). Another human trial showed that a species of mushroom, *Agaricus bisporus,* can contribute to a delicious taste known as kokumi taste, gives a mouthful sensation, and is rich in taste, which makes it the best among other mushroom species (43). The species of mushroom described in Table 2.

Various species of edible mushrooms are involved in the modification of the human defensive system and exhibit biological activities such as antioxidant, antimicrobial, and many other medical benefits (Table 2). Various phytochemical compounds are present in many edible mushrooms, specifically flavonoids, terpenoids, alkaloids, polysaccharides, and polyphenols, which exhibit multiple biological activities against human ailments (44). Due to nutritional values, various edible mushrooms gain attention for consumption. Different studies showed that the phytochemical composition of edible mushrooms has the potential to protect and fight against various human disorders, including oxidative stress, Parkinson’s disease, Alzheimer’s disease, neuroinflammation, and prion disease, through the mechanism of these phenolic components’ activity (23). Various studies showed that many edible mushrooms have medicinal properties and exhibit multiple therapeutic properties (45). Different phytochemical and bioactive compounds in edible mushrooms have various physiological properties, which are divided into the following groups:

### 3.1 Anticancer activity

**Figure 3:**
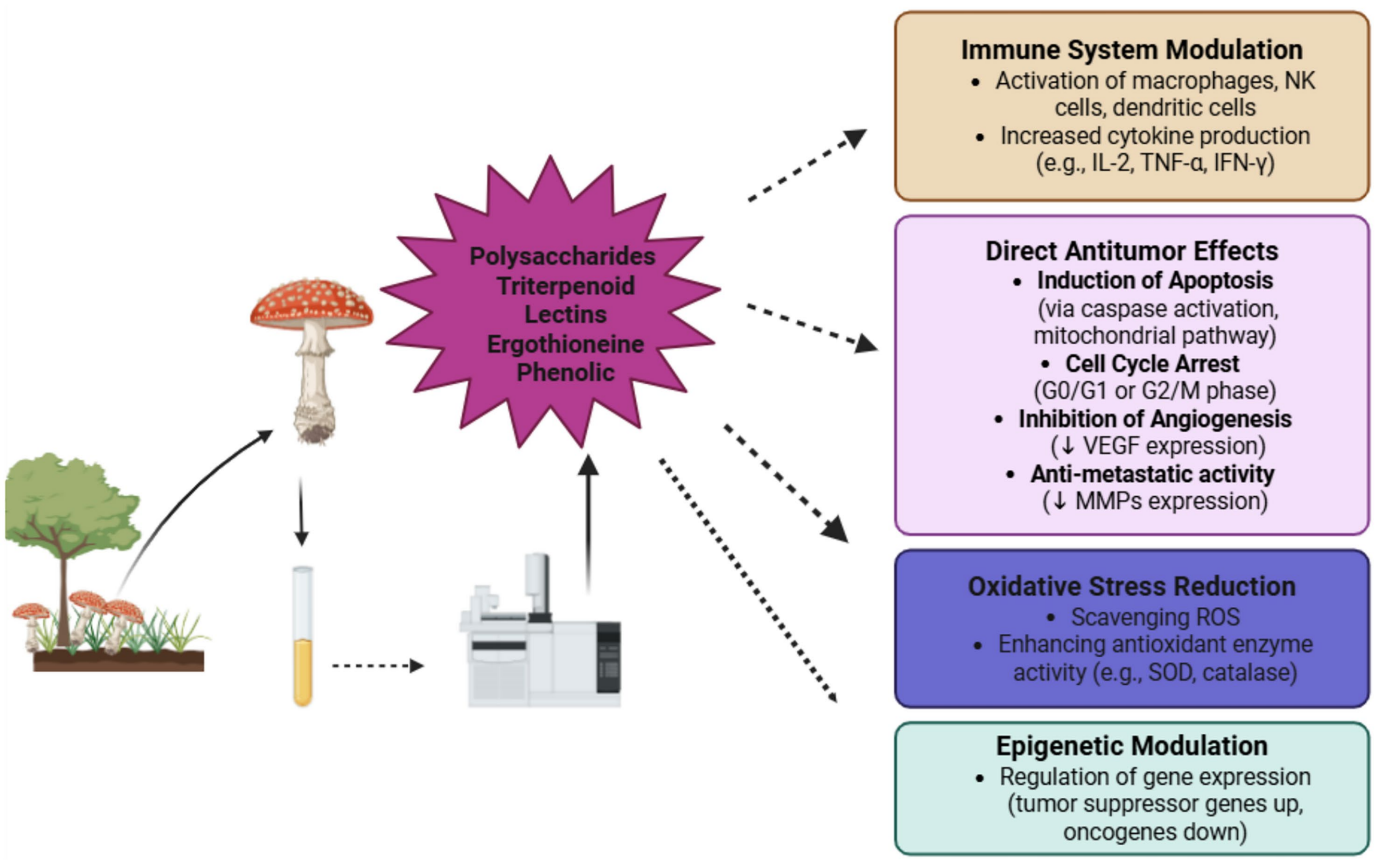
Edible mushrooms in anticancer activities.

Various edible mushrooms, including *Tremella mesenterica*, *Agaricus brasiliensis*, *Pleurotus ostreatus*, *Sanghuangprous vaninii,* and other species of mushrooms that possess potential medicinal properties due to their rich composition of bioactive phytochemicals as mentioned in section 2 (46). The extracts of these mushrooms include chemicals such as phenolics, flavonoids, polysaccharides (particularly *β*-glucans), terpenoids, sterols, and lectins, which display substantial anti-inflammatory, antioxidant, immunomodulatory, and anticancer properties. Phenolic chemicals contained in these mushrooms (such as gallic acid, ferulic acid, and caffeic acid) have an important role in carcinogenesis prevention by reducing free radical formation and altering signaling pathways involved in cell proliferation and death. These drugs have demonstrated effectiveness in lowering the incidence and development of breast, colorectal, cervical, and liver cancers by inducing apoptosis, limiting angiogenesis, and blocking metastasis *in vitro* and *in vivo* models (12, 44). The phytochemical compounds, particularly terpenoids, have the potential to play an antioxidant role by reducing oxidative stress and scavenging free radicals, as shown in *vitro* studies (Figure 3). These actions help protect DNA from oxidative damage and enhance cellular antioxidant defense mechanisms. Most edible mushrooms are rich in *β*-glucans, which are non-digestible polysaccharides known to modulate the immune system by activating macrophages, natural killer (NK) cells, and T-cells. In the context of cancer, *β*-glucans can suppress tumor cell proliferation and induce apoptosis through immune-mediated mechanisms, as both *in vitro and in vivo* studies. Specifically, β-glucans in mushrooms such as *Pleurotus ostreatus* and *Agaricus brasiliensis* are effective in suppressing breast cancer cell lines by modulating cytokine production and enhancing immune surveillance in *in vivo* studies. Furthermore, β-glucans contribute to cholesterol reduction by binding bile acids in the intestine, leading to increased cholesterol excretion and improved lipid profiles. This lipid-lowering effect has been supported by both animal and human trials. Numerous studies support the anticancer potential of edible mushrooms through these multiple mechanisms, highlighting their role in preventing tumor growth, reducing inflammation, and enhancing antioxidant defenses (44, 47, 48).

### 3.2 Antidiabetic activity

**Figure 4:**
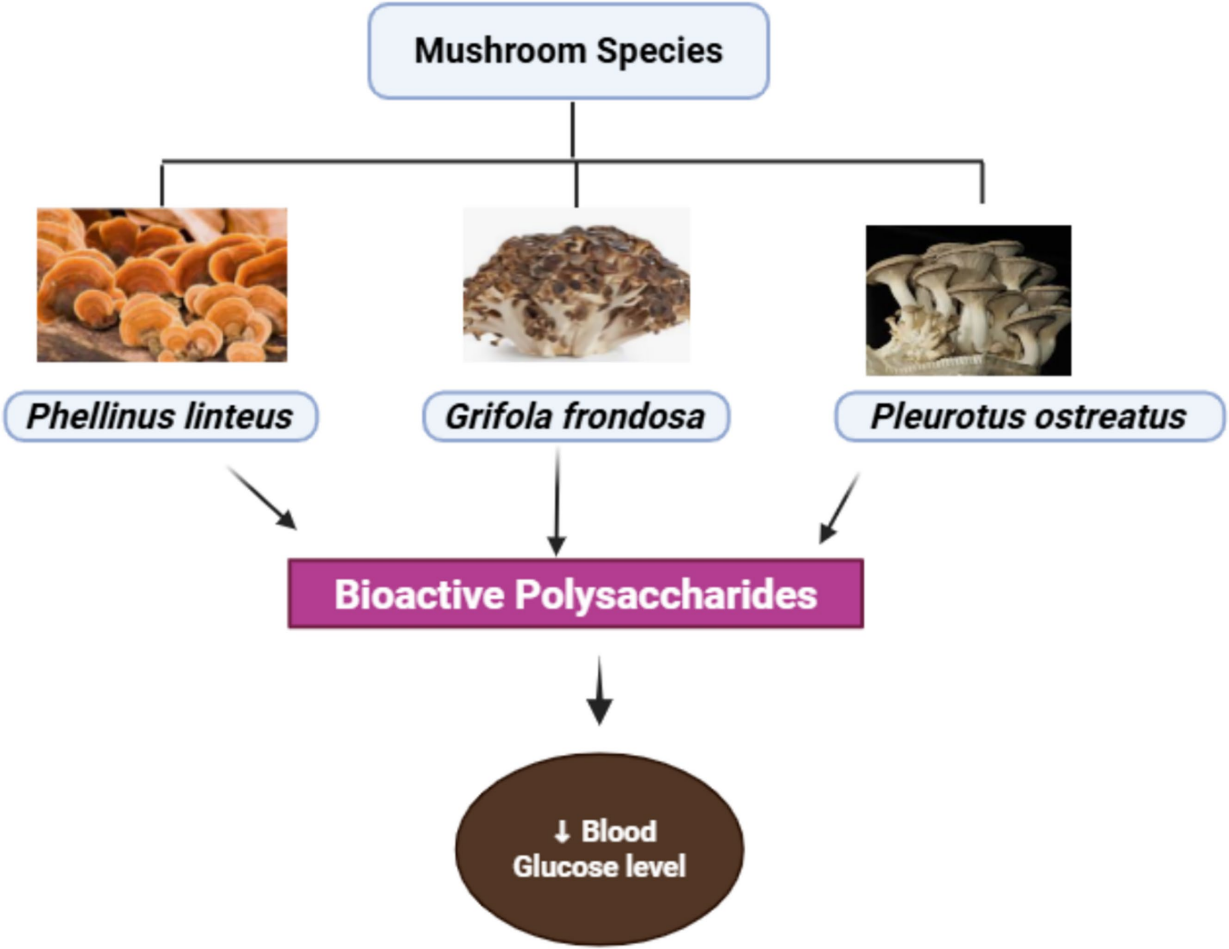
Hypoglycemic effect of mushroom species.

Different species of edible mushrooms have different bioactive compounds, including polysaccharides that play a vital role in improving human health (49). Results from studies suggest that most edible mushrooms have bioactive compounds that help in the regulation of glucose levels against diabetic conditions. *Pleurotus* has been shown to possess phenolic compounds that have potential hypoglycemic activity. A recent *in vivo* experimental study showed that oral administration of a mushroom, particularly *P. eryngii* extract, to hyperglycemic mice significantly regulates the glucose level. This result indicated that *P. eryngii* extract can potentially manage hyperglycemic problems in people (50). Aqueous extract of various mushrooms, including *P. pulmonarius* and *P. citrinopileatus, can strongly minimize* insulin sensitivity in diabetic mice due to the potential anti-diabetic activity of polysaccharides, as shown in Figure 4. Previous *in vivo* studies showed that various kinds of mushrooms, including the genus of Agaricus that have anti-glycemic properties (51). An *in vivo* experimental model showed that the rate of diabetes type II has been significantly reduced in diabetic rats as they get the powder of A*. bisporus* (52, 53). In another *in vivo* experimental study, the results indicated that *A. sylvaticus* possesses the potential to significantly minimize the risk of type 1 diabetes, HDL level, blood glucose level, and fat accumulation to an extent (45). A*. blazei* also has the potential to improve diabetic conditions by enhancing insulin secretion and possesses a protective effect against various human disorders, supported by human trials. Other *in vivo* experimental trials showed that insulin levels are enhanced and the risk of diabetes reduced by oral administration in diabetic rats, and after 28 days of experiments, significant results were observed. Results from all these *in vivo* studies suggest that most of the species of mushrooms have the potential to manage diabetes in both diabetic rats and diabetic people (54, 55).

### 3.3 Cardiovascular and hypertension activity

Various species of mushrooms are involved in cardio-protective activity, including *Agaricus bisporus*. The result of an *in vivo* experimental study showed that bioactive compounds, particularly peptides in mushrooms, have the potential to protect from cardiac problems (43). Various types of mushrooms have the potential to reduce hypertension by suppressing the activity of the hypertension Angiotensin-converting enzyme (ACE). Many mushrooms, including the genera *Pleurotus*, *Granola fronds*, *Agarics Bosporus* and other mushrooms, have bioactive compounds that can act as ACE inhibitors and protect from cardiovascular diseases, and therapeutic and pharmaceutical applications of these mushrooms have proven that they are an excellent source to reduce the risk of hypertension, as supported by an *in vitro* studies (56). Several *in vitro* studies showed that various mushrooms have antihypertensive constituents, particularly peptides and triterpenes which have the potential to protect from high blood pressure and eventually minimize the risk of hypertension by suppressing the activity of the ACE and renin angiotensin-aldosterone system. Another *in vitro* studies the results showed that *A. Bosporus* has anti-hypertensive activity due to presence of amino acids and peptides that possess the potential to protect from cardiovascular diseases by inhibiting the activity of ACE (57). In another *in vitro* study, it was proven that *P. pulmonarius mycelium* can enhance the anti-hypertensive activity by inhibiting the effect of the hypertension-causing enzyme ACE. The extract of various mushrooms, specifically *H. marmoreus* has the potential to reduce blood pressure in hypertensive rats as it contains an ACE inhibitor (58).

### 3.4 Immune-function activity

Various species of mushrooms, particularly *Pleurotus ostreatus* and their lectin extracts, can enhance the activity of the defensive system in humans, as demonstrated in *in vitro* and *in vivo* studies (19). A wide range of bioactive compounds in edible mushrooms act as antimutagenic and can modulate the immune system and its functions. These edible mushrooms have multiple bioactive compounds, particularly beta-glucan, which promote anti-inflammatory, immunomodulatory, and antioxidant activity. These phytochemical compounds help to provide a protective mechanism against various human ailments. The bioactivity of phenolic constituents of edible mushrooms can modulate and stimulate the T and B cells, which help to protect from cancerous cells (59). Different mushrooms have various phytochemical compounds, particularly polysaccharides that possess scavenging and antioxidant activity to help against immune diseases, as shown by *in vitro* studies. These phenolic components have the potential to protect from free radicals, proliferation, and growth of cancerous cells through *in vitro* antitumor activity as discussed in section 2 (60).

### 3.5 Antiviral activity

**Figure 5:**
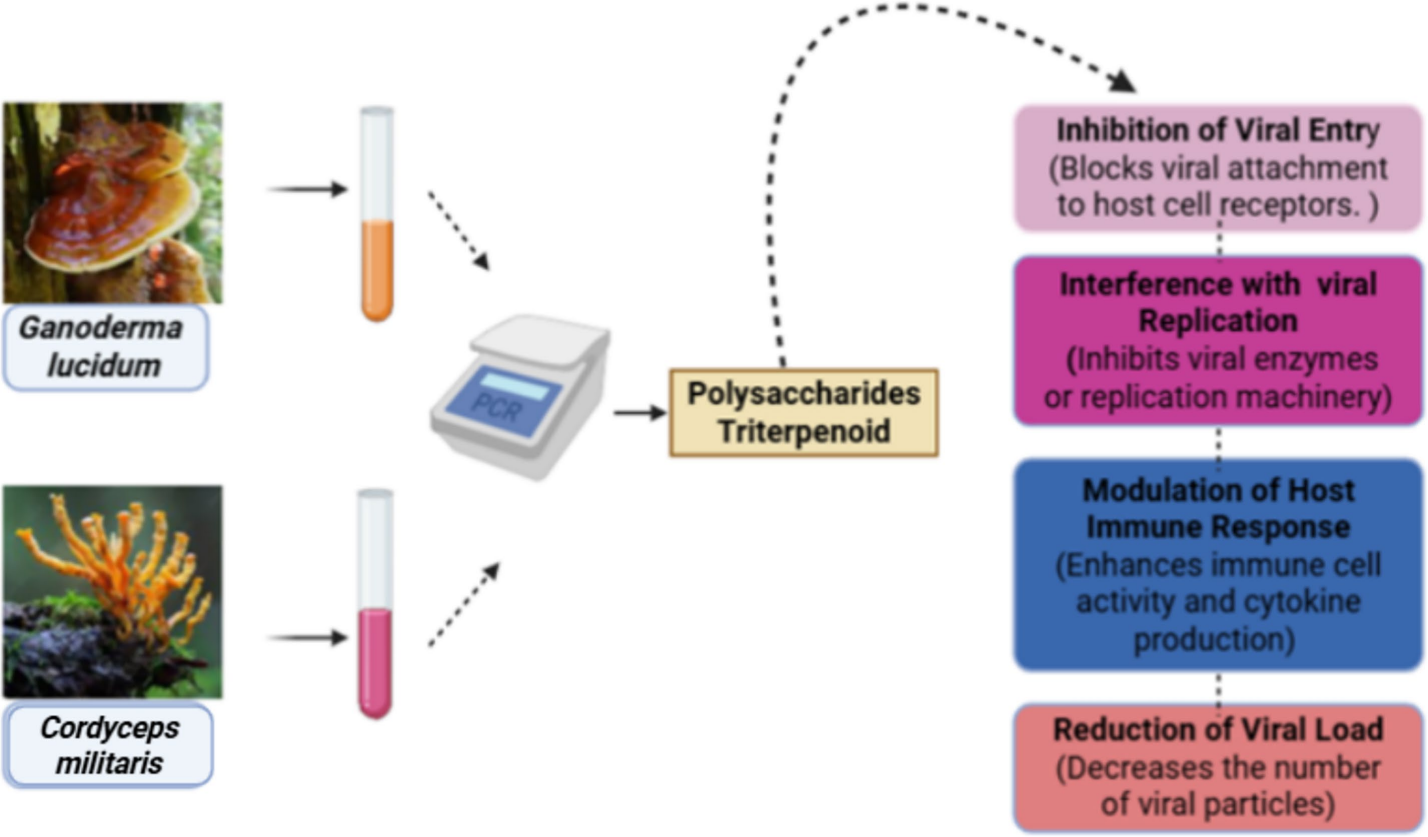
Anti-viral activity of the mushroom species.

The results of different studies showed that various mushrooms have anti-viral activity against several viruses that affect human health, including herpes simplex virus (HSV), influenza, and human immunodeficiency virus (HIV). Several mushroom extracts have antiviral compounds that help prevent viruses and provide a defensive mechanism against these viruses. Various edible mushrooms have medicinal properties and protect human health. The number of edible mushrooms, including *Ganoderma colossus* (rich in triterpenoids, polysaccharides, and phenolic compounds), *Lentinus edodes* (containing lentinan, *β*-glucans, and ergosterol), and *Ganoderma lucidum* (abundant in triterpenoids, ganoderic acids, and polysaccharides), has shown *in vitro* anti-viral properties in their phytochemical composition, which protects from attack of various viral diseases in humans. A wide range of mushroom species, including *Ganoderma lucidum* has demonstrated *in vitro* potential to protect from Herpes simplex virus (HSV) due to the presence of anti-viral bioactive compounds, including its triterpenoids (especially ganoderic acid B and C1) and polysaccharides, which possess the ability to fight against this virus (61). Several mushrooms are involved in protection from viruses, one of them is *Phellinus igniarius*, which has shown *in vitro* activity against the influenza virus and protects from this virus by the potential anti-viral mechanism through its bioactive components such as polyphenols, hispidin derivatives, and polysaccharides (7). Hepatitis virus is very dangerous to human health, as it affects the human body badly. Different species of mushrooms, including *Lentinula edodes*, *Antrodia camphorata,* have shown *in vitro* anti-viral activity due to the presence of polysaccharides, triterpenoids, and adenosine derivatives in the chemical composition of mushrooms. Various *in vitro* studies also demonstrated biological activities of various mushrooms due to the presence of phytochemical compounds, particularly polysaccharides have the potential to protect from COVID-19 and other viruses (11). Additionally, several species of mushrooms and their extracts, including *Agaricus blazei* (rich in *β*-glucans, ergosterol, and lectins) and *Hypsizygus marmoreus* (containing polysaccharides, sterols, and phenolic compounds), have demonstrated *in vitro* activity against foot-and-mouth disease virus due to anti-viral activity of polysaccharides, sterols, and other bioactive compounds, as shown in Figure 5 (62).

### 3.6 Anti-neurodegenerative activity

Several edible mushrooms, such as *Sarcodon scabrosus*, *Grifola frondosa*, *Pleurotus giganteus,* and *Hericium erinaceus* have numerous medicinal activities, particularly boosting cognitive functions. Several phytochemical compounds and their constituents in many mushrooms have the potential to protect against various human ailments (44). Various mushrooms such as *Hericium erinaceus* and *Sarcodon scabrosus* have the potential to improve cognitive functions, brain and nerve health (63). Various human trials showed that a few mushrooms possess more potential for improvement of neuro-health properties, one of them is *H. erinaceus*, which exerts beneficial effects on neuro-health and the nervous system (64). *H. erinaceus* has various bioactive compounds that improve the nervous system by stimulating the synthesis of nerve growth factor (NGF), which helps to maintain and regulate the sensory nervous system. The neuroprotective effect of *H. erinaceus* helps to protect from neurodegenerative disorders, including anxiety, depression, and Alzheimer’s disease, by improving cognitive functions. The therapeutic application of *H. erinaceus* also suppresses the progression of cognitive impairment and many other brain-associated disorders (65). The other biological application of *H. erinaceus* is to improve sleep quality through the action mechanism of neuroprotective bioactive compounds. An *in vivo* experimental study over 30 days showed that the powder form of *H. erinaceus* exerts potential effects on the brain when administered orally in mice (66).

### 3.7 Anti-obesity and hyperlipidemic activity

Various extracts of different mushroom species, such as Ophiocordyceps sinensis and *Pleurotus eryngii* used to treat obesity due to the presence of polysaccharides and *β*-glucan in water extracts and many other bioactive compounds (67). Various studies demonstrated that mushrooms can reduce hyperlipidemia in obese people, such as *Lentinula edodes,* which has the potential to protect from obesity and possesses anti-hyperlipidemic activity. A recent *in vivo* study showed that *L. edodes* is significantly involved in the anti-obesity effect when induced with a high-fat diet in obese mice by reducing fat accumulation and triglyceride levels (68). Another vivo study indicated that *L. edodes* has many anti-obesity components, and its high dose is linked with the prevention of obesity. Previous studies showed that high levels of triglycerides in the liver are higher than in adipose tissues. A specific content, Eritadenine, is present in *L. edodes,* which plays a significant role in the prevention of obesity by reducing dyslipidemia and phosphatidylcholine (PC) in the liver (69).

A major phospholipid, PC, is mainly involved in the secretion of lipoproteins in the liver. Eritadenine can significantly lower the triglyceride levels in plasma, but it is also involved in increasing triglyceride concentration in the liver. Later on, the study revealed that the administration of Eritadenine with choline supplementation did not involve increasing triglyceride concentration in the liver, and the deficiency of PC was managed by adding choline chloride. Another useful species of mushroom, genus *Pleurotus* helps to protect from gaining weight, and its beneficial effects involve in reduction of hyperlipidemia, as mentioned *in vivo* trial (70).

An important component of *β*-glucans in *Pleurotus sajor-caju* helps to protect from oxidative stress and obesity in obese mice by the action mechanism of bioactive components, which involves in inhibition of adipocytes and lipolysis. Further *in vivo* studies indicated that *P. eryngii* has important components, such as polysaccharides, which possess a hypolipidemic effect and are involved in the reduction of lipid levels in fat-loaded mice. During 6 weeks of feeding rats with *P. eryngii* indicated that it has strong potential in improving antioxidant and hepatic lipase properties in hepatic (71). The results of various studies showed that the consumption of edible mushrooms is associated with reducing the incidence of various cardiovascular diseases. Numerous bioactive compounds help to protect against increasing lipid levels. A wide range of edible mushrooms are involved in biological activities to protect against various human ailments, including high triglycerides and hypoglycemia. The antioxidant and anti-inflammatory properties of phytochemical compounds in edible mushrooms have potential to provide benefits for management against a number of health complications (72, 73).

A wide range of edible mushrooms is enriched with health benefits due to the presence of polysaccharides and antioxidant activity. Recent *in vitro* studies showed that phenolic components in mushrooms and their extracts can act as antioxidants (27). The methanol extract of edible mushrooms and fruiting bodies showed significant results against various human disorders by the action mechanism of phenolic compounds, antioxidant activity, and phenolic acid, as mentioned *in vitro* study. Few mushrooms, including *Gloeophyllum sepiarium* have more antioxidant properties by reducing oxidative stress and providing benefits to the human body due to the presence of linoleic acid (46). Edible mushrooms comprise many important components, one of which is ergosterol, which plays an important role in antioxidant activity and acts as a precursor for the production of vitamin D. The methanolic extract of various mushrooms has inhibitory activity against various oxidative agents and possesses the ability to scavenge free radicals and hydroxyl radicals. Several *in vitro* studies indicated that most of the mushrooms such as *Agrocybe cylindracea*, *H. erinaceus*, D*. indusiata*, L*. edodes,* and many other beneficial mushrooms, acquire antioxidant activity (17, 38).

## 4 Industrial uses of edible mushrooms

A numbers of edible mushrooms possess nutraceutical properties, which provide numerous benefits to human health by improving body functioning and lowering the incidence of various diseases (8, 56). The phenolic compounds in edible mushrooms and their derivatives comprise significant amounts of benzoic acid, phenolic acids, and cinnamic acid, which play a major role against various physiological conditions. The number of phenolic components, such as caffeic acid, p-hydroxybenzoic acid, gallic acid, p-coumaric and, and polysaccharides, galactans, *β*-glucan, xylans, and other phenolic content, makes the edible mushrooms unique and beneficial (21, 74–76). Increasing consumption of mushrooms and pharmaceutical applications is well known for treating various diseases. Rich bioactive profiles of various mushroom species provide wide industrial uses. For its antioxidant, anti-inflammatory, and taste-enhancing qualities, *Russula griseocarnosa* finds usage in food, pharmaceutical, and cosmeceutical sectors (77). *Agaricus* species support hepatoprotective, anticancer, and biotechnological applications, including *A. Blazei* and *A. Bisporus* (78). Food taste, skincare, and metabolic health benefit from *Pleurotus eryngii* and *Lentinula edodes* (79). While *Ganoderma frondosa* and *L. edodes* help with immune regulation and soil improvement, *Lignosus rhinoceros* is prized for neuroprotective and skin-repair products (21, 80). Meat substitutes, effects on cholesterol, and environmental advantages abound from *Pleurotus* spp. (81). Support liver health, metabolic control, and fermentation technologies. *Antrodia salmonea* and *Sanghuangprous vaninii* support (21, 64, 82). At last, *Pleurotus citrinopileatus* is a cosmeceutical and nutritional gourmet mushroom with pharmacological and medicinal potential (83). Industrial values of some important compounds originated from different mushrooms shown in Table 3.

**Table 3:** Industrial usages of edible mushrooms.

Mushroom species	Industrial usages	Form of preparation	References
*Russula griseocarnosa* (Russula)	In food industry Natural food additives Meat substitutes and flavor enhancers In the Pharmaceutical Industry Antioxidant and anti-inflammatory formulations Antitumor or adjunctive cancer therapy supplements In the Cosmeceutical Industry Anti-inflammatory products Anti-oxidant rich formulation	Ethanolic or aqueous extracts Dried Powder	(77)
*Agaricus blazei* (Sun Mushroom); *Agaricus bisporus* (Button Mushroom)	In the Pharmaceutical and Nutraceutical Industry Hepatoprotective formulation Anti-tumor therapy Blood glucose and lipid regulators Functional Food Industry In the Cosmeceutical Industry Anti-inflammatory products Skin care products In the Agricultural and Biotechnological Applications Biofertilizers Waste valorization Mycoremediation potential:	Hot water extracts Fermented products dried powder encapsulated forms	(78)
*Pleurotus eryngii* (King Oyster Mushroom), *Lentinula edodes* (Shiitake Mushroom)	In the Agricultural and Environmental Applications Organic farming Mycoremediation In the Cosmeceutical Industry Skin care products In the Pharmaceutical & Nutraceutical Industry Anti-tumor therapy Blood glucose and lipid regulators Cholesterol-lowering products Antioxidant and anti-inflammatory products In the Food Industry Traditional cuisine Food flavoring	Fermented extracts mycelial powder Aqueous/ethanolic extracts Dried mushroom	(79)
*Lignosus rhinoceros* (Tiger Milk Mushroom)	In the Pharmaceutical & Nutraceutical Industry Anti-asthmatic and anti-inflammatory agents Antitumor potential Neuroprotective products In the Cosmeceutical Industry Anti-aging and skin-repair formulations Wound healing In the Functional Food and Beverage Industry Health drinks and tonics Mushroom-infused foods In the Biotechnology and Research Cultivation and fermentation Patent development	Hot water and methanol extracts Lyophilized powder Cultured mycelia	(21, 80)
*Ganoderma frondosa* (Maitake), *Lentinula edodes* (Shiitake)	In the Pharmaceutical & Nutraceutical Industry Antitumor and immune-boosting agents Metabolic health support Anti-viral and anti-inflammatory agents In the Functional Food Industry Health beverages and extracts Maitake-infused products Flavor enhancer In the Environmental and Agricultural Use Organic fertilizer and soil enhancer Mycoremediation potential	Fruiting body extracts Polysaccharide-rich extracts Dried forms	(92)
*Pleurotus* spp. (Oyster Mushrooms)	In the Food and Functional Food Industry Eatable mushrooms Meat alternative Fermented Products In the Pharmaceutical and Nutraceutical Applications Cholesterol-lowering agents Anti-diabetic supplements Neuroprotective benefits In the Environmental & Agricultural Applications Animal feed additive Biodegradation of plastics Mycoremediation	Whole dried Fermented products Polysaccharide extracts	(81)
*G. lucidum* (Reishi / Lingzhi)	In the Pharmaceutical and Nutraceutical Industry Immunomodulatory Effects Anticancer Adjunct Therapy Hepatoprotective & Cardiovascular Health Neuroprotective and Anti-aging Effects In the Functional Food and Beverage Industry Medicinal mushroom teas, coffee blends Supplement drinks and health tonics In the Cosmeceutical Industry Skin care products Anti-aging products	Hot water extracts Spore powder Encapsulated extracts	(17)
*Antrodia salmonea* (stout camphor fungus)	In the Pharmaceutical and Nutraceutical Industry Liver-protective and Antioxidant Formulations Diabetes and Metabolic Syndrome Management Anticancer and Chemopreventive Properties In the Biotechnology and Cultivation Industry Liquid fermentation and solid-state cultivation In the Functional Food and Beverage Industry medicinal mushroom teas capsules, tonics	Mycelial biomass Ethanol extracts Capsule formulation	(21, 82)
*Sanghuangprous vaninii* (Sanghuang mushroom)	In the Pharmaceutical and Nutraceutical Industry Immunomodulatory and Anti-inflammatory Supplements Liver-protective and Antioxidant Formulations Diabetes and Metabolic Syndrome Management In the Functional Food and Beverage Industry Medicinal teas and drinks Fermented mushroom products In the Biotechnology and Cultivation Industry Solid-state and liquid fermentation systems	Solid-state fermented powder Aqueous/ethanol extracts	(64)
*Pleurotus citrinopileatus* (Golden Oyster Mushroom)	In the Food and Functional Food Industry Meat substitute Edible gourmet mushroom Nutrient-rich ingredient In the Pharmaceutical and Nutraceutical Industry Cholesterol-lowering supplements Antioxidant products Anti-inflammatory and hepatoprotective formulations Cosmeceutical Applications Skin-care additives Natural pigment and antioxidant	Whole dried Water/ethanol extracts Powdered ingredient	(83)

## 5 Toxicity of different species of mushrooms

Various species of mushrooms are poisonous to health, even if they are edible or inedible. Number of edible mushrooms can be poisonous due to various reasons, such as inappropriate processing of excessive consumption, and particular toxic material in mushrooms, including trehalose content. In the recent study, it has been shown that T*richoloma equestre is* considered an edible mushroom, but is reported as poisonous due to the adverse effects of specific toxic content (84). Another study proved that a species of mushroom *A. phalloides,* causes toxication due to the presence of toxic content amatoxin. *A. muscaria* is also recommended as a toxic mushroom, which causes intoxication due to the adverse and toxic effects of ibotenic acid (IBO), and its consumption causes hallucinogenic effects. The experimental study identified that *Clitocybe amoenolens* also exerts a toxic effect when administered to rats (71). Different mushrooms cause poisoning to different extents according to the level of toxicity in them. Various are cytotoxic and Myotoxic; therefore, they are inhibited to consumed so they cannot cause nephrotoxicity, neurotoxicity, gastroenteritis, autonomic toxicity, and many other poisoning syndromes (85). Several studies reported that about 35 species of mushrooms contain amatoxins, which contribute to intoxication in the human body (74, 79). Mushrooms are well-known for their capacity to bioaccumulate a wide range of elements from their growing environment, including heavy metals (e.g., lead, cadmium, mercury, and arsenic) as well as emerging metals like as platinum (Pt), palladium (Pd), and rhodium. This provides a health danger, particularly when mushrooms are grown in polluted locations such as industrial zones, mining sites, or roadsides. Prolonged use of mushrooms containing these toxic components can cause major health problems, such as neurotoxicity, renal damage, and even cancer (86). While some metals, like as copper and zinc, are required in tiny amounts, high doses can be hazardous. Furthermore, the presence of radionuclides such as cesium-137 in wild mushrooms from areas damaged by nuclear fallout is a source of worry. Continuous monitoring and regulation of metal contamination in edible mushrooms is required to maintain consumer safety. Table 4 summarizes the risks due to the presence of undesired substances such as heavy metals and emerging metals (Pt, Pd, Rh, etc.) in mushrooms, especially those grown in contaminated areas.

**Table 4:** Toxic metal contaminants and associated risks in edible mushrooms.

Category	Undesired substances	Sources of contamination	Risk to human health	Remarks	References
Heavy metals	Lead (Pb), Cadmium (Cd), Mercury (Hg), Arsenic (As)	Industrial pollution, mining areas, sewage sludge, and traffic emissions	Neurotoxicity, kidney damage carcinogenicity developmental disorder	Accumulate quickly in mushrooms due to their high bioaccumulation potential.	(83, 86)
Emerging metals	Platinum (Pt), Palladium (Pd), Rhodium (Rh)	Vehicle catalytic converters, industrial emissions	potential for allergic reactions cytotoxicity	Increasing concern due to rising environmental levels	(68, 93)
Soil Contaminants	Copper (Cu), Zinc (Zn), Nickel (Ni)	Fertilizers, pesticides, industrial waste	Gastrointestinal issues, liver/kidney damage (in high amounts)	Essential in small amounts; toxic at high concentrations	(43, 89)
Radionuclides	Cesium-137 (Cs-137), Strontium-90 (Sr-90)	Nuclear accidents (e.g., Chernobyl, Fukushima)	Radiation exposure cancer bone marrow suppression	Wild mushrooms in some regions still show radioactive contamination	(57, 84)

## 6 Conclusion

Edible mushrooms like *Agaricus bisporus*, *Pleurotus ostreatus*, *Lentinula edodes*, and *Ganoderma lucidum* are known for having a lot of bioactive compounds that are good for your health. These include *β*-glucans, phenolic acids (gallic acid, caffeic acid, and ferulic acid), flavonoids, terpenoids, sterols (like ergosterol), polysaccharides, lectins, and essential amino acids. These compounds exhibit numerous therapeutic effects, including strong antioxidant activity, immunomodulatory properties, anti-cancer effects, and actions that protect the liver, combat viruses, and kill microorganisms. These health benefits make edible mushrooms interesting functional foods with a lot of promise in the pharmaceutical, nutraceutical, and cosmeceutical businesses. They can also improve gastrointestinal health, regulate the immune system, and combat oxidative stress, making them a good fit for preventive healthcare plans. Still, the fact that dangerous species like *Amanita phalloides* (which has lethal amatoxins) and *Clitocybe amoenolens* (which has neurotoxic ibotenic acid) are present shows how important it is to properly identify, educate, and regulate the usage of mushrooms. Future research should focus on the isolation, characterization, and mechanistic understanding of mushroom-derived bioactive compounds to unlock their full therapeutic potential. Expanding their incorporation into general diet practices could serve as a sustainable, natural approach to enhancing human health and reducing disease risk.
